# Niosomes as promising approach for enhancing the cytotoxicity of *Hemimycale* sp. total crude extract supported with in-silico studies

**DOI:** 10.1038/s41598-024-52918-3

**Published:** 2024-01-31

**Authors:** Asmaa Abo Elgoud Said, Basma Khalaf Mahmoud, Abdelrahman M. Helmy, Nada M. Mohamed, Eman Zekry Attia, Mamdouh Nabil Samy, Usama Ramadan Abdelmohsen, Mostafa A. Fouad

**Affiliations:** 1 Department of Pharmacognosy, Faculty of Pharmacy, Minia University, Minia, 61519 Egypt; 2 Department of Pharmacognosy, Faculty of Pharmacy, Deraya University, Universities Zone, New Minia City, 61111 Egypt; 3 Department of Pharmaceutics and Pharmaceutical Technology, Faculty of Pharmacy, Deraya University, Minya, Egypt; 4 Pharmaceutical Engineering and 3D Printing (PharmE3D) Lab, Division of Molecular Pharmaceutics and Drug Delivery, College of Pharmacy, The University of Texas at Austin, Austin, TX 78712 USA; 5 Pharmaceutical Chemistry Department, Modern University for Technology and Information (MTI), Cairo, Egypt

**Keywords:** Metabolomics, Natural products

## Abstract

The crude extract of *Hemimycale *sp. marine sponge was evaluated as a cytotoxic drug against different cell lines; whereas it exhibited promising selective activity toward the breast cancer cell line only with IC_50_ value 199.6 ± 0.00512 µg/ml. Moreover, its cytotoxic activity against the breast cancer cell line was reevaluated upon forming total extract-loaded niosomes. This revealed an IC_50_ value of 44.35 ± 0.011128 µg/ml, indicating the potential contribution of niosomes in boosting cell penetration and activity as a result. Owing to highlight the bioactive constituents responsible for the cytotoxic activity, metabolomics profiling of *Hemimycale *sp. was performed using liquid chromatography coupled with high-resolution electrospray ionization mass spectrometry (LC-HR-ESI-MS) revealing tentative identification of phytoconstituents clusters like as, diterpenes, sesterterpenes and sterols. Additionally, the cytotoxic activity of the crude extract was explained on the molecular level, whereas the dereplicated compounds were evaluated in silico against the Epidermal Growth Factor Receptor tyrosine kinase (EGFR). The sesterterpenoid derivatives phorbaketal A acetate **(12)** and secoepoxy ansellone A **(13)** together with mycalol-522 **(17)** showed the best binding energy.

## Introduction

The ocean, the “mother of life,” covers more than 70% of the earth’s surface and is extremely diverse in terms of ecology, chemistry, and biology, including everything from microbes to vertebrates. This diversity has served as a source for rare chemical compounds with promising therapeutic applications. The study of the marine ecosystem to uncover countless complex and innovative chemical entities is emphasized by new developments in drug discovery from natural sources^1^. Marine species are still relatively underutilized. Many creatures are made up of chemicals and materials with intriguing traits and features, which serve as an inspiration source for the creation of new medically focused drugs^2^. There are a number of dozen marine natural products that are undergone clinical or preclinical trials for treatment of cancer, and development of marine compounds as potential medicines is gaining enormous interest. Didemnin B was the first marine natural product to enter human clinical trials against cancer and paved the way for a multitude of therapeutic candidates isolated from marine organisms^3^.

Family Hymedesmiidae is a valid source of numerous cytotoxic compounds that have been isolated, such as zarzissine alkaloid, which was previously isolated from *Phorbas paupertas* sea sponge and exhibited cytotoxic activity against various cell lines, including murine leukaemia and nasopharyngeal carcinoma^4^. Moreover, a new guanidine alkaloid Ptilomycaline (A) was the first compound identified from *Hemimycale *sp.*,* exhibited cytotoxic activity against the leukaemia P-388 cell line^4^. The delivery of phytochemicals *via* nano carriers is gaining prominence in the treatment of cancer because it can improve bioavailability, target tumor cells specifically, and boost cellular absorption, all of which can lead to a large reduction in dosage and a consistency in therapeutic outcomes^5–7^. Because they are mostly made of non-ionic surfactants and cholesterol, niosomes are amphiphilic vesicular nano carriers that can efficiently encapsulate natural products with a variety of physicochemical characteristics^8^. Recent research highlighted the role of niosomes in augmenting the cytotoxic action of a variety of natural product extracts, including propolis, green tea, and Carum extracts^9–11^.

In the light of the aforementioned data, the total crude extract of *Hemimycale* sp. was evaluated for its cytotoxic activity against four cancerous cell lines and a normal cell line. Furthermore, formulation of crude extract-loaded niosomes were carried out, and the cytotoxic activity of the prepared extract-loaded niosomes was then evaluated again against breast cancer cell line. To highlight the phytoconstituents, responsible for the cytotoxic activity, metabolomics analysis of the crude extract was performed. Likewise, in silico molecular docking simulation were evaluated to clarify the suggested mechanisms against the breast cancer cell line.

## Material and methods

### Specimen collection and preparation

*Hemimycale* sp. sponge material was collected from a long patchy reef, Ahia Reefs, at the north of Hurghada (Red Sea) and then the freeze dried materials was cut into small pieces and extracted by maceration at room temperature with a 50/50 mixture of dichloromethane and methanol which were obtained from El-Nasr Company for Pharmaceuticals and Chemicals, Egypt. The extracting solution was concentrated under reduced pressure, afforded the crude extract (0.6 g) which was used for further investigations.

#### Preparation of extract-loaded niosomes:

For the formation of extract-loaded niosomes, the crude extract of *Hemimycale* sp. sponge material was thoroughly mixed with ethanol by sonication for 30 min. The ethanolic mixture was then filtered through 45 µm diameter filter to collect the ethanolic solution of the sponge extract (29 mg/ml). The sponge-loaded niosomes were prepared by the thin film hydration test^12^. In a 100 ml round-bottomed flask, 219 mg of span 20 and 105 mg of cholesterol were dissolved in 8 ml ethanol, and then 2 ml of the ethanolic solution of the extract was added. The flask content was evaporated at 65 °C under vacuum in a rotary evaporator (Heidolph rotary evaporator, Germany) rotating at 80 rpm, until the precipitation of a dry thin film on the bottom of the flask. The flask was placed in the freezer for 30 min. Following that, the dry film was hydrated by adding 10 ml of deionized water to the flask. The flask contents were mixed at 62 °C for 140 min by rotating at 120 rpm. The obtained sponge-loaded niosomes dispersion was then divided into two parts. The first part was stored in the refrigerator (4 °C) to maintain the niosomes’ original large size and was referred as sponge-loaded large niosomes (SLN). The second part was sonicated in a bath sonicator for 20 min to create sponge-loaded small niosomes (SSN) and then transferred to the refrigerator^13^. The same procedures were used to produce unloaded small niosomes (USN).

#### Size analysis and Zeta potential measurement

Samples of SLN, SSN and USN were analyzed for their particle size in terms of the average volume diameters by photon correlation spectroscopy using particle size analyzer Dynamic Light Scattering (DLS) (Zetasizer Nano ZN, Malvern Panalytical Ltd, United Kingdom) at fixed angle of 173° at 25 °C. Samples were analyzed in triplicate. The same equipment was used for the determination of zeta potential.

### Cytotoxic activity

The cytotoxic activity of the crude extract of *Hemimycale* sp. was carried out against various cancer cell lines; hepatocellular carcinoma (HepG2), prostate carcinoma (Pc3), colon carcinoma (HCT116), and human breast cancer (Mcf7), together with normal cell line from lung (wi38). All tested cell lines were obtained from Sigma-Aldrish, Germany. In which, a 96 well tissue culture plates were inoculated with 1 × 10^5^ cells/ml (100 µl/well) and incubated at 37 °C for 24 h to develop a complete monolayer sheet. After forming a confluent sheet of cells, growth medium was decanted from 96 well microtiter plates, and the cell monolayer was washed twice with wash media. Two-fold dilutions of the tested sample were made in RPMI medium containing 2% serum (maintenance medium), and 0.1 ml of each dilution was tested in different wells, with three wells serving as controls and receiving only maintenance medium. The plate was incubated at 37 °C and checked for any physical signs of toxicity, such as partial or complete loss of the monolayer, rounding, shrinkage, or cell granulation. MTT solution (5 mg/ml in PBS) (BIO BASIC CANADA INC) was prepared, and 20 µl of the solution was added to each well and shaken at 150 rpm for 5 min to thoroughly mix the MTT into the media. The media was incubated for 1–5 h at 37 °C, 5% CO_2_ to allow the MTT to be metabolized then, was dumped to remove any residue, and the formazan (MTT metabolic product) was resuspended in 200 µl DMSO and shaken at 150 rpm for 5 min to thoroughly mix the formazan into the solvent. The optical density was calculated at 560 nm and subtracts background at 620 nm which was correlated with cell quantity.

### Metabolomics profiling

The chemical profiling of crude extract of *Hemimycale* sp. was performed for the first time, using LC-HR-ESI-MS for dereplication purposes. The detected compounds were tentatively identified by employing macros and algorithms that coupled MZmine with online and in-house databases. Prior to dereplication, MZmine’s algorithm was used to predict molecular formulas, which employs a combination of empirical techniques such as isotope pattern matching. With the help of the Marinlit and DNP databases for marine natural products, known compounds were tentatively identified using positive and negative mode electrospray ionization spectral data at a MW tolerance of 10 ppm^14^.

### Docking study

The X-ray crystal of Epidermal Growth Factor Receptor tyrosine kinase (PDB 1M17) was retrieved from the Protein Data Bank then ligand and water molecules which were not involved in the interaction were removed. The protein structure was corrected and 3D protonated at the default pH and temperature with electrostatic calculation according to GB/VI algorithm and a cutoff of 15 Å. The tested compounds chemical structures were drawing using ChemDraw^®^ Ultra 12.0 then pasted into MOE^®^ as smiles. The compounds database was protonated and energy minimized using MMFF94x force field at gradient 0.1 kcal mol^−1^ Å^−1^. Their partial charges were calculated at the same force field without constraints. The molecular docking protocol was validated before commencing the actual docking procedure by co-crystallized ligand self-docking to get the lowest RMSD. The used docking protocol implemented triangle matcher, London dG and GBVI/WSA dG as placement, rescoring 1 and rescoring 2 algorithms, respectively.

## Results and discussion

### Niosomes characterization

The niosomal formulation of the crude extract of *Hemimycale* sp. was successfully formed through using the thin film hydration method, and the characteristic qualities of the prepared niosomes including particle size and the zeta potential are displayed in Table 1. For the colloidal stability of the niosomes dispersion, the zeta potential is considered one of the most critical factors. The zeta potential of USN (*c.a.* 312.6 ± 14.88 mv) was acceptable^15^. By loading the crude extract, the sponge extract components reduced the zeta potential of the formulated niosomes to be − 14.3 ± 2.86 mV and − 13.4 ± 1.04 mV for SLN and SSN, respectively, enhancing the likelihood of aggregation occurrence. However, the particle size analysis and the morphological properties of the SLN and SSN revealed no signs of aggregation. The average size of the sponge-loaded large niosomes (SLN) was 4650 ± 807.8 nm. The sonication process reduced the size of the sponge-loaded small niosomes (SSN) and the unloaded niosomes (USN) profoundly to 294.0 ± 1.5 nm and 312.6 ± 14.88 nm.

**Table 1:** The average volume diameter and the zeta potential of the large and small sponge-loaded niosomes formulations versus unloaded niosomes.

Sample	Size (nm ± SD)	Zeta potential (mV ± SD)
SLN	4650 ± 807.8	− 14.3 ± 2.86
SSN	294.0 ± 1.5	− 13.4 ± 1.04
USN	312.6 ± 14.88	− 23.5 ± 0.97

### Cytotoxic activity

**Figure 1:**
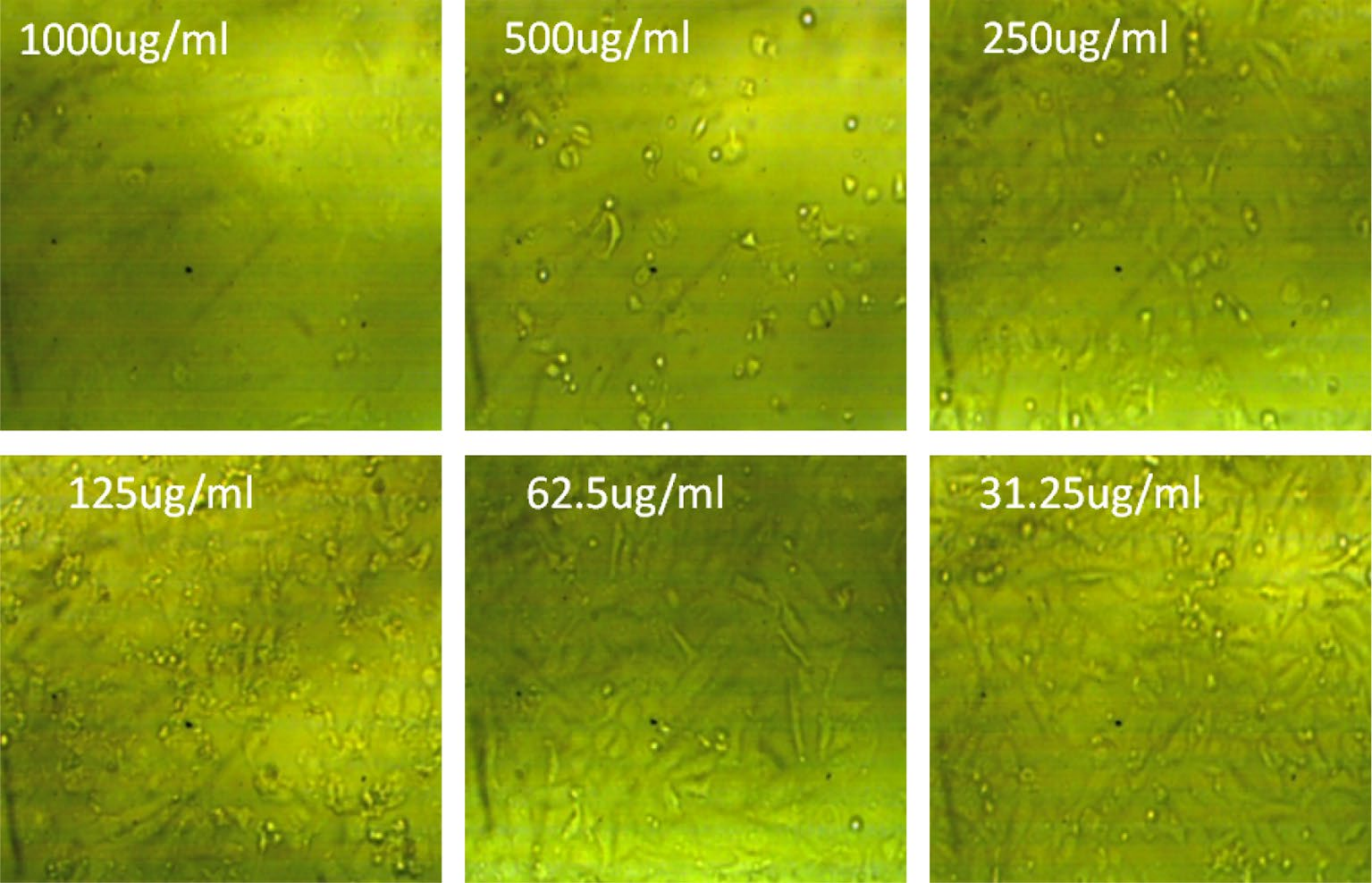
The effect of crude extract on breast cancer cell line at different concentration.

**Figure 2:**
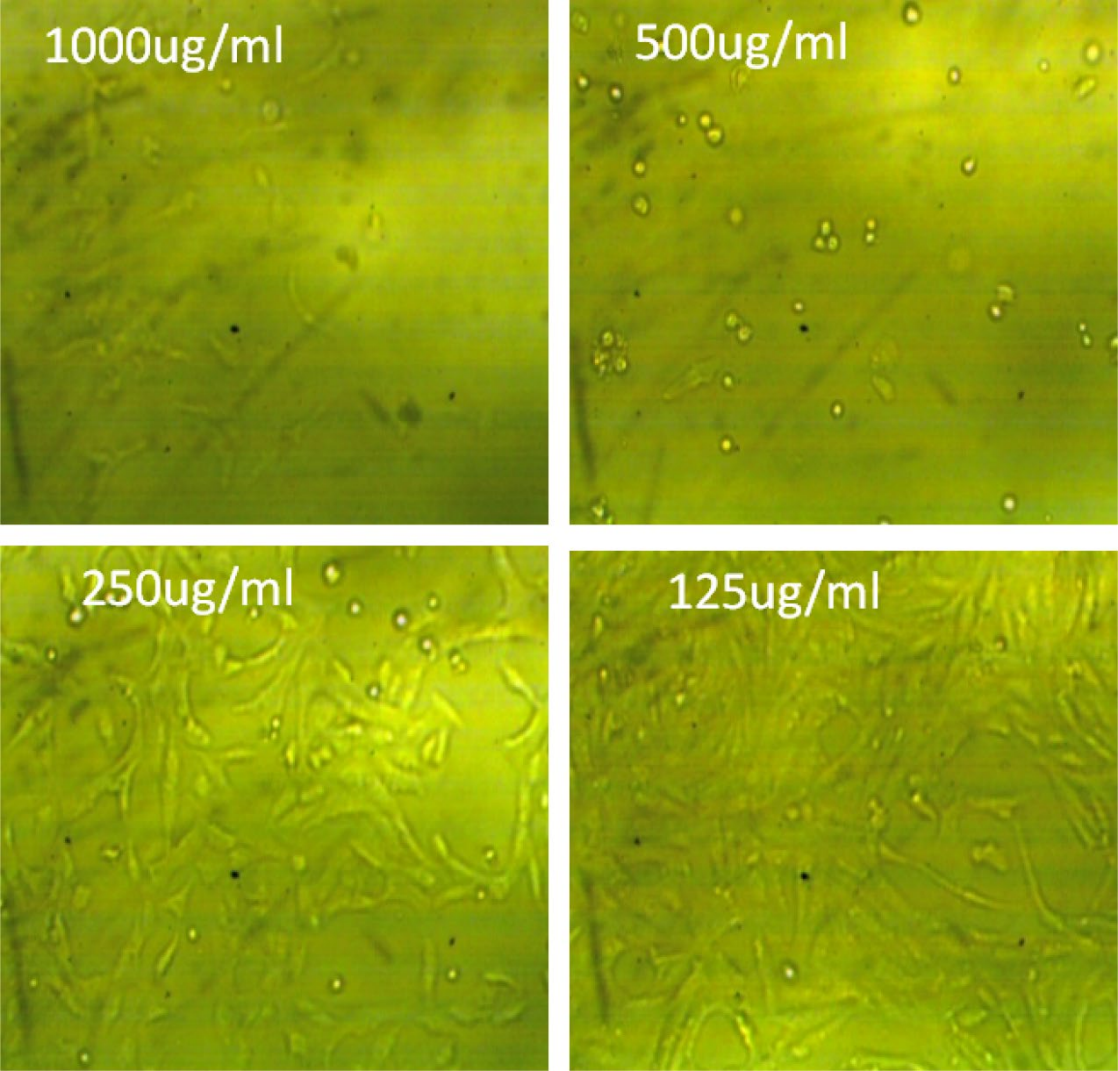
The effect of crude extract on normal cell line (Wi 38) at different concentration.

The cytotoxic activity of the Red sea sponge, *Hemimycale* sp. crude extract was evaluated against HepG2, Pc-3, HCT-116, Mcf-7 cell lines and the normal cell line wi-38. The crude extract showed moderate to weak selective activity against only breast cancer cell line with IC_50_ value of 199.6 ± 0.00512 µg/ml and against normal cell line (Wi 38) with IC_50_ value of 367.4 ± 0.00472 µg/ml revealing 1.84 index of selectivity. Figures 1 and 2 shows the effect of crude extract on breast cancer and normal cell lines at different concentrations, respectively.

On the other hand, the cytotoxic activity evaluation of the formulated SLN, SSN and USN samples against breast cancer cell line, showed IC_50_ values of 241.3 ± 0.00628, 44.35 ± 0.011128 and 183.5 ± 0.00670 µg/ml, respectively. This finding demonstrates that the small size of niosomes had a significant impact on the penetration of sponge extract into cancer tissues and supports previously reported data on an inverse association between niosomes’ size and penetration degree^9,16^.

### Metabolomics profiling

Metabolomics profiling of the crude extract has resulted in the characterization of multivariate classes of components whereas, diterpenes and sesterterpenes are the most abundantly expressed ones. Firstly hamigerans and phorbasins diterpenes were dereplicated like as, the mass ion peak at *m/z* 413.0857 for the suggested molecular formula C_19_H_25_BrO_5_ was dereplicated as Hamigeran L **(1)** and it was obtained from *Hamigera trangensis*^17^. Phorbasin compounds were also detected as the mass ion peak at *m/z* 317.2026 with the molecular formula C_20_H_28_O_3_ was dereplicated as Phorbasin **A (2)** and the mass ion peak at *m/z* 335.2136 for the proposed molecular formula C_20_H_30_O_4_ was dereplicated as Phorbasin B **(3),** both were previously isolated from *Phorbas* sp.^18,19^. Additionally, Phorbasin H **(4)** and phorbasin H1 **(5)** similarly have been detected, the former with the mass ion peak at *m/z* 361.2291, in agreement with the molecular formula C_22_H_32_O_4_, whereas, the latter with the mass ion peak at *m/z* 305.2383, in agreement with the molecular formula C_20_H_32_O_2_. Both phorbasin H and H1 were previously isolated from *Phorbas *sp.^4,20,21^. In addition, Phorbasin I **(6)** and phorbasin I1 **(7)** have been dereplicated from the mass ion peak at *m/z* 405.2548, in agreement with the molecular formula C_24_H_36_O_5_, for the former one, while the latter with the mass ion peak at *m/z* 305.2383, in agreement with the molecular formula C_20_H_32_O_2_. Phorbasin I was isolated from *Phorbas gukulensis,* while phorbasin I1 was isolated from *Phorbas *sp.^4,20,21^. Another phorbasin compound, K **(8)**, was also detected with the mass ion peak at *m/z* 337.2287 and molecular formula C_20_H_32_O_4_ and it was also previously isolated from *Phorbas* sp.^21^.

In addition, the aforementioned diterpenes, sesterterpenoid compounds were also dereplicated. In this respect, the mass ion peak at *m/z* 399.2442, in agreement with the molecular formula C_25_H_34_O_4_, was dereplicated as alotaketal A **(9)** and/or phorbaketal A**(10)**. Alotaketal A was isolated from *Hamigera* sp.^22^*,* while phorbaketal A was isolated from *phorbas* sp.^23^. Phorbaketal A acetate **(11)** was also dereplicated with the mass ion peak at *m/z* 485.2829 in agreement with the molecular formula C_25_H_34_O_4_ and was previously isolated from *Phorbas* sp.^24^ Also, the mass ion peak at *m/z* 457.2523, in consistent with the molecular formula C_27_H_36_O_6_, was identified as secoepoxy ansellone **A (12),** ansellone **F (13)**, and/or ansellone **G (14)**, which were previously isolated from *Phorbas* sp.^25,26^.

**Figure 3:**
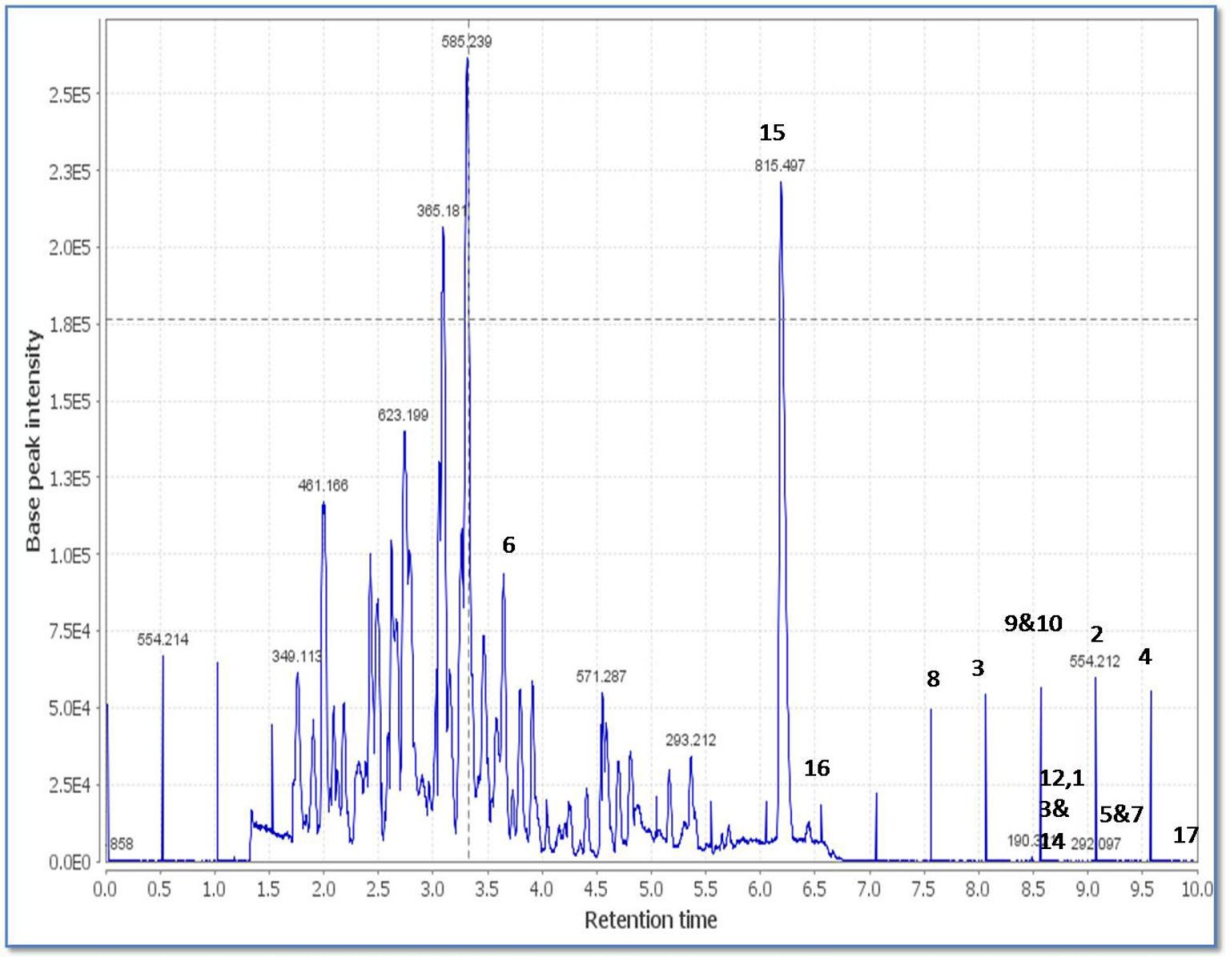
Total ion chromatogram of crude extract of *Hemimycale* sp.

**Figure 4:**
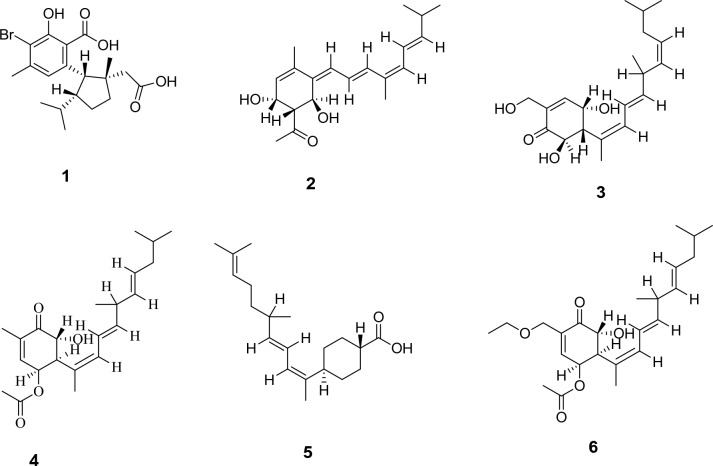
Chemical structure of dereplicated compounds **1**–**6**.

**Figure 5:**
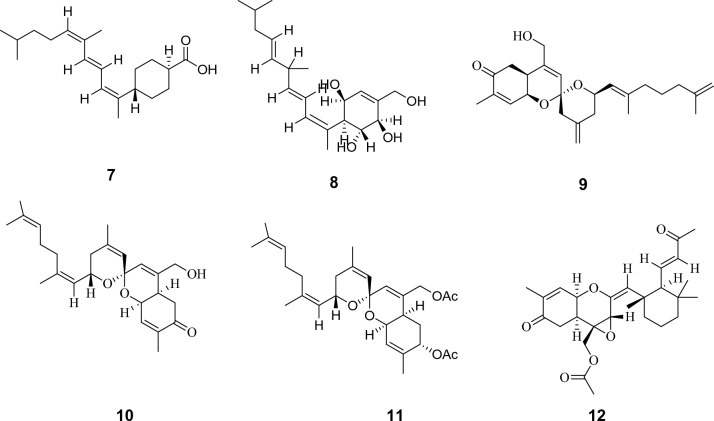
Chemical structure of dereplicated compounds **7**–**12**.

**Figure 6:**
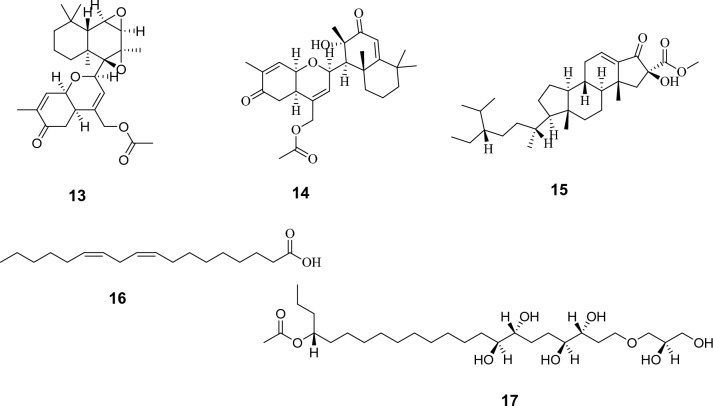
Chemical structure of dereplicated compounds **13**–**17**.

Moreover, the mass ion peak at *m/z* 473.3534 corresponding to the predicted molecular formula C_30_H_48_O_4_ was dereplicated as phorbasterone D **(15)**, a steroid component previously isolated from *Phorbas* sp.^27^, whereas linoleic acid **(16)** and mycalol-522 **(17)** compounds were also dereplicated, the former with the mass ion peak at *m/z* 281.2408, in agreement with the molecular formula C_18_H_32_O_2,_ whereas the latter with the mass ion peak at *m/z* 523.3738, in consistent with the molecular formula C_27_H_54_O_9_. Linoleic acid was isolated from *Hemimycale arabica*^28^*,* while mycalol-522 was previously isolated from *Hemimycale topsenti*^29^. Many of the dereplicated compounds as phorbasins diterpenes have a good reputation of their substantial potency and selectivity together with the sesterterpenoid, phorbaketal A against cancer cell lines^4,21,23^. Total ion chromatogram of the total extract of the sponge under study were shown in Fig. 3 and the chemical structure of the dereplicated metabolites is depicted in Figs. 4, 5 and 6.

### In silico molecular docking simulation

The achieved cytotoxic activity of the crude extract was needed to explain on molecular level. Therefore, the dereplicated compounds were in silico evaluated against the Epidermal Growth Factor Receptor tyrosine kinase (EGFR). EGFR normally regulates the cell proliferation and is found among the cell membrane. However, its overexpression was reported in many types of cancers such as lung, breast and kidney carcinoma^30,31^. Structurally, EGFR consists of three domains; extracellular receptor-like, trans membranal and intracellular kinase domains^32^. EGFR is established to be in a monomeric form during its resting state which upon activation, Lys721 forms ion-pair with the conserved Glu738 to interact with the ATP phosphate groups^33,34^. Its ATP-binding domain in the intracellular region possesses a conserved amino acid sequence with 39 residues locate near the ATP binding site among which Leu718, Val726, Ala743, Met793, and Leu844 showed abundant ligand interaction^35^.

**Figure 7:**
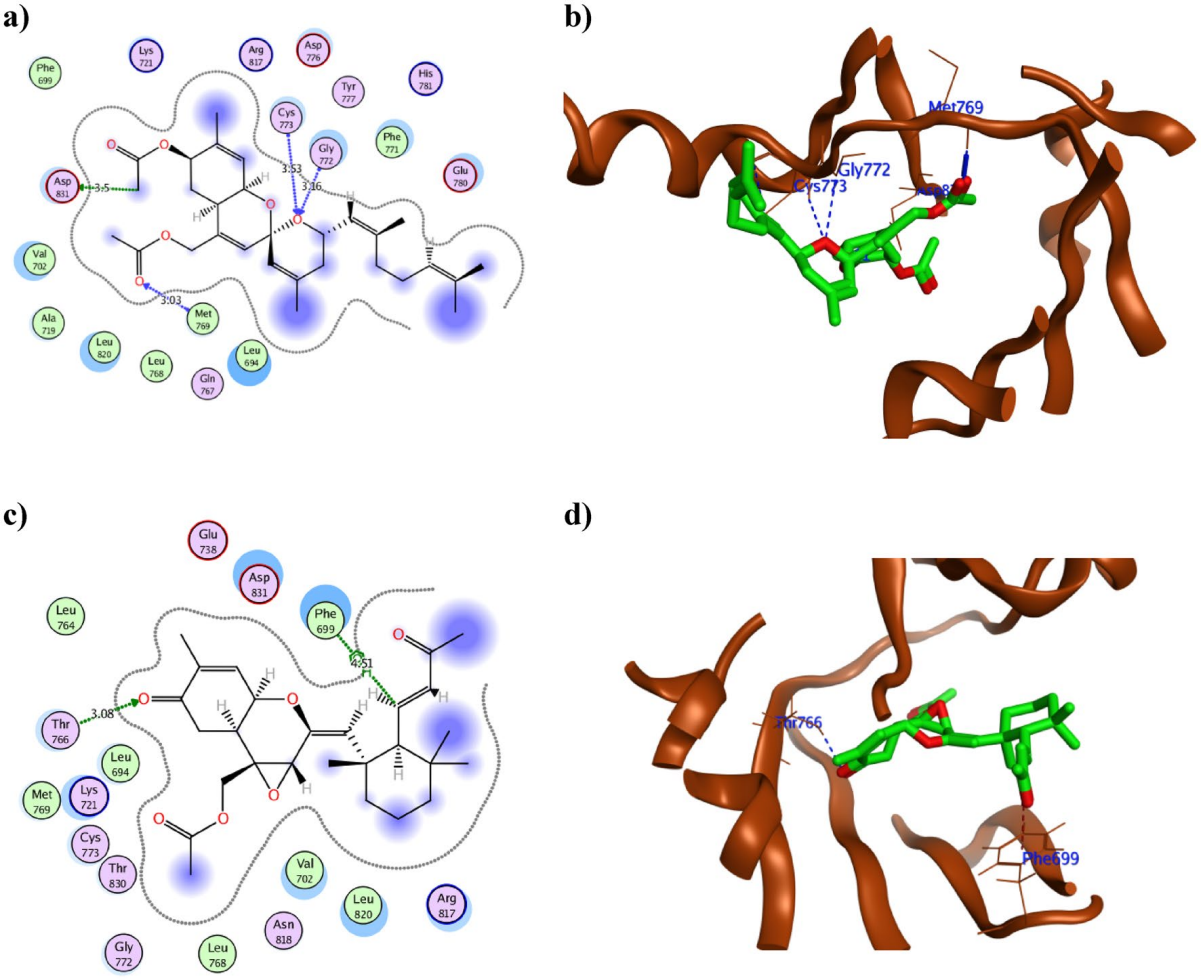
The 2D and 3D presentations of the binding conformation of phorbaketalA acetate **12** (**a**, **b**), secoepoxyAnselloneA **13** (**c**, **d**) against EGFR (PDB: 1M17) appeared as green stick model with the hydrogen and hydrophobic bonds displayed as green and red dotted lines, respectively.

**Figure 8:**
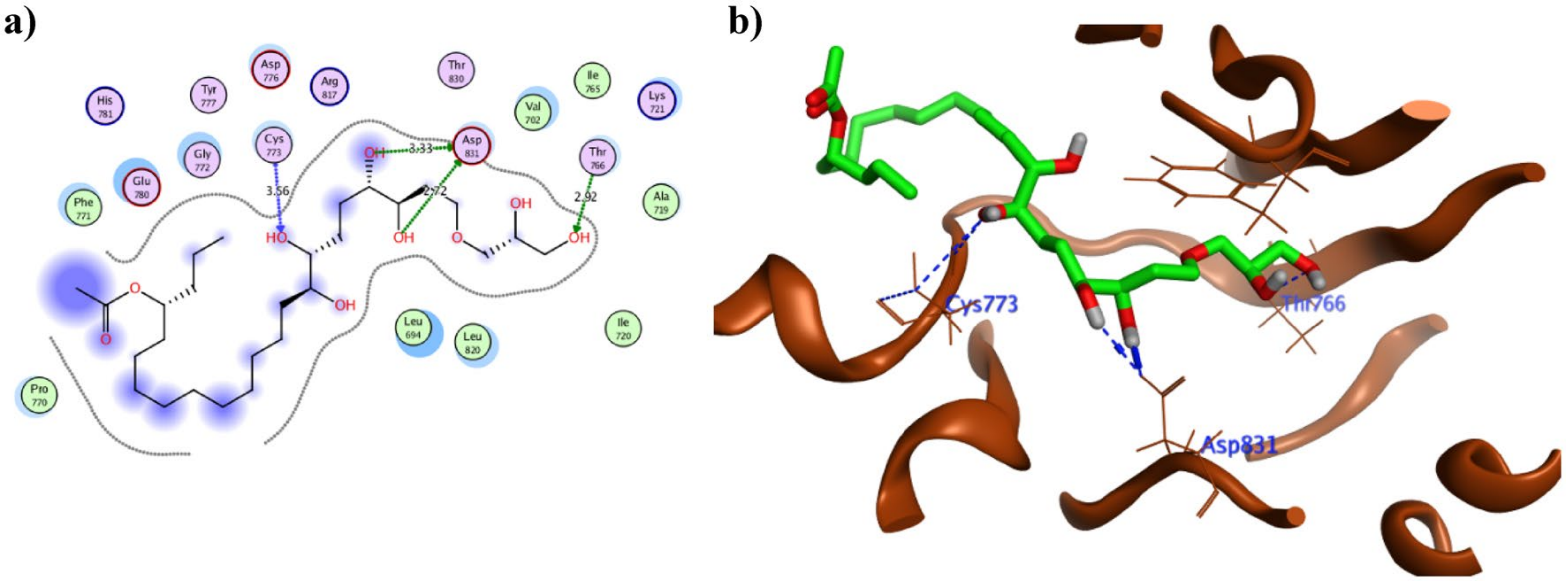
The 2D and 3D presentations of the binding conformation of mycalol-522 **17**(**a**, **b**) against EGFR (PDB: 1M17) appeared as green stick model with the hydrogen and hydrophobic bonds displayed as green andred dotted lines, respectively.

**Figure 9:**
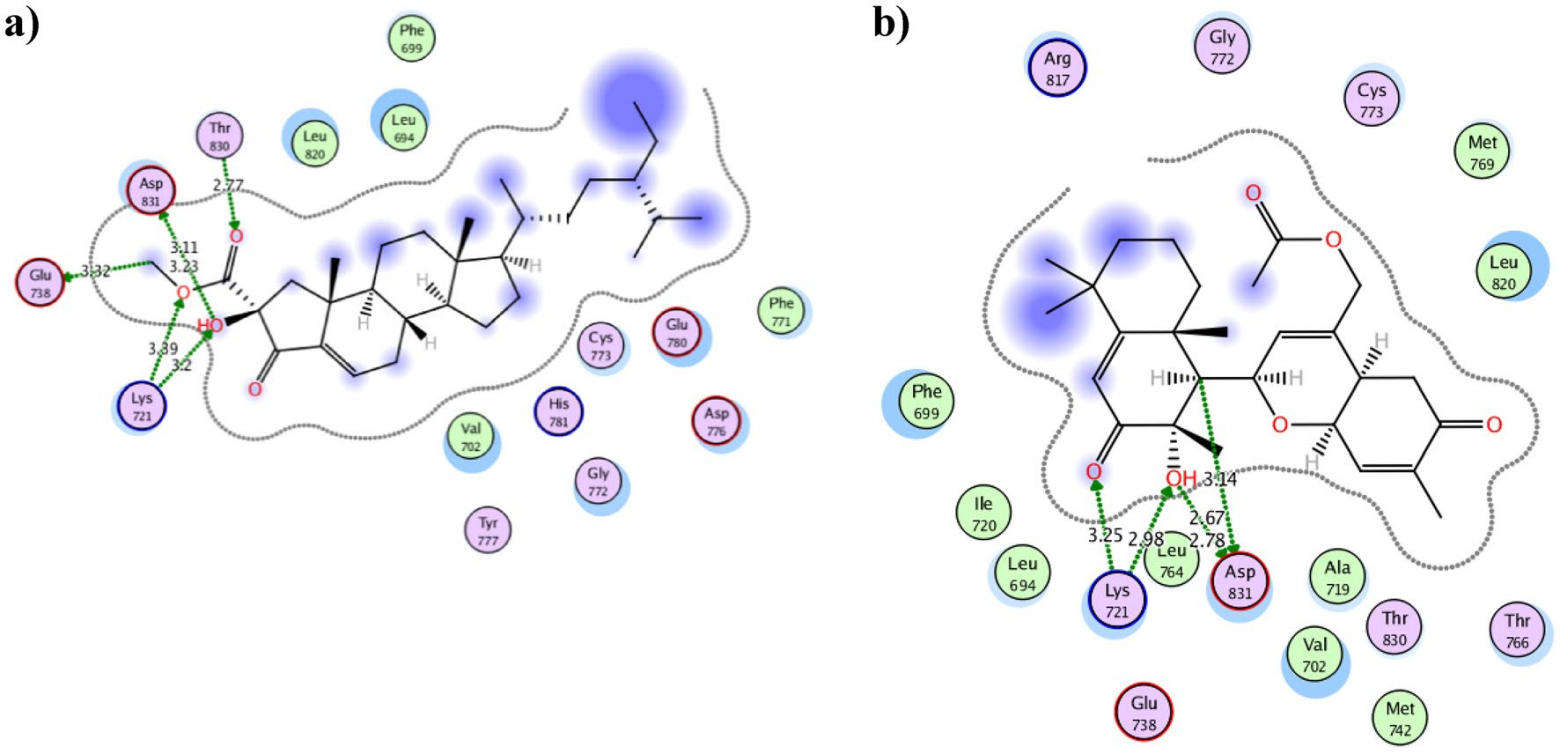
The 2D interactions of phorbasterone D **10** (**a**) and ansellone G **15** (**b**) using EGFR (PDB: 1M17) showing bond distances in Å.

The attained molecular docking results of the 17 dereplicated compounds were presented at Table 2 with their 2D and 3D conformations of the best fitted derivatives were demonstrated at Figs. 7, 8 and 9. The molecular docking protocol was validated before starting the actual simulation by re-docking the co-crystallized ligand **AQ4** using different docking algorithms. The best algorithm achieved RMSD 1.27 Å conserving the same interactions.

**Table 2:** The molecular docking results of the 17 dereplicated compounds of *Hemimycale* sp. crude extract using EGFR (PDB ID: 1M17) in comparison to the co-crystallized ligand **AQ4**.

Compound	Binding energy score (Kcal/mol)	Ligand Interacting moiety		Kinase			Type of interaction	Distance in Å	Interaction energy (Kcal/mol)
				Interacting moiety	Amino acid residue				
**AQ4**	− 9.54	C19	45	O	Gln	767	H-donor	3.15	− 1.00
		N2	44	N	Met	769	H-acceptor	2.70	− 2.10
**1**	− 7.22	O	23	O	Gln	767	H-donor	2.82	− 1.40
		O	25	SG	Cys	751	H-donor	3.57	− 1.10
		O	25	OG1	Thr	830	H-acceptor	3.00	− 2.10
		O	22	NZ	Lys	721	ionic	3.11	− 3.80
**2**	− 7.87	O	8	NZ	Lys	721	H-acceptor	3.62	− 0.60
**3**	− 8.36	O	23	OD2	Asp	831	H-donor	3.12	− 2.10
		O	21	NZ	Lys	721	H-acceptor	3.01	− 6.80
**4**	− 8.65	C	12	SD	Met	742	H-donor	4.02	− 0.40
		O	23	SD	Met	742	H-donor	3.38	0.30
		O	24	OD2	Asp	831	H-donor	2.80	− 1.50
		O	24	N	Asp	831	H-acceptor	3.31	− 0.10
**5**	− 8.77	C	20	OD2	Asp	831	H-donor	3.44	− 0.20
		O	25	OD1	Asp	831	H-donor	2.77	− 2.60
		O	25	OD2	Asp	831	H-donor	2.99	− 2.10
		O	24	NZ	Lys	721	H-acceptor	2.76	− 4.50
		O	24	CA	Asp	831	H-acceptor	3.54	− 0.20
		O	25	NZ	Lys	721	H-acceptor	3.07	− 2.60
**6**	− 7.32	O	23	OD1	Asp	831	H-donor	2.67	− 2.60
		O	21	OD2	Asp	831	H-donor	2.99	− 2.10
**7**	− 7.81	O	21	NZ	Lys	721	H-acceptor	2.88	− 7.90
		O	22	NZ	Lys	721	H-acceptor	2.92	− 11.20
		O	21	NZ	Lys	721	Ionic	2.88	− 5.30
		O	22	NZ	Lys	721	Ionic	2.92	− 5.00
**8**	− 7.66	O	21	NZ	Lys	721	H-acceptor	2.94	− 12.30
		O	22	NZ	Lys	721	H-acceptor	2.95	− 3.00
		O	21	NZ	Lys	721	Ionic	2.94	− 4.90
		O	22	NZ	Lys	721	Ionic	2.95	− 4.80
**9**	− 8.03	C	12	OE2	Glu	738	H-donor	3.20	− 0.40
		O	23	OD1	Asp	831	H-donor	3.05	− 0.50
		O	23	OD2	Asp	831	H-donor	3.00	− 1.20
		O	24	OD2	Asp	831	H-donor	3.02	− 2.40
		O	23	NZ	Lys	721	H-acceptor	2.98	− 6.00
**10**	− 9.19	C	7	OE2	Glu	738	H-donor	3.32	− 0.50
		O	33	OD1	Asp	831	H-donor	3.11	− 3.00
		O	33	OD2	Asp	831	H-donor	3.23	− 0.40
		O	32	OG1	Thr	830	H-acceptor	2.77	− 0.50
		O	33	NZ	Lys	721	H-acceptor	3.20	− 1.20
		O	34	NZ	Lys	721	H-acceptor	3.39	− 0.70
**11**	− 8.41	C	11	OD2	Asp	831	H-donor	3.42	− 0.60
		O	26	N	Met	769	H-acceptor	3.37	− 0.70
		O	27	OG1	Thr	766	H-acceptor	3.22	− 0.40
**12**	− 9.90	C	39	OD2	Asp	831	H-donor	3.50	− 0.20
		O	26	CA	Gly	772	H-acceptor	3.16	− 0.30
		O	26	N	Cys	773	H-acceptor	3.53	− 0.20
		O	33	N	Met	769	H-acceptor	3.03	− 3.90
**13**	− 9.94	O	30	OG1	Thr	766	H-acceptor	3.08	− 0.30
		C	9	6-ring	Phe	699	H-pi	4.51	− 0.70
**14**	− 8.94	C	2	SD	Met	742	H-donor	3.80	− 0.40
		C	10	O	Arg	817	H-donor	3.62	− 0.30
		C	17	OD2	Asp	831	H-donor	3.35	− 0.20
**15**	− 9.11	C	24	OD2	Asp	831	H-donor	3.14	− 0.80
		O	31	OD1	Asp	831	H-donor	2.67	− 1.90
		O	31	OD2	Asp	831	H-donor	2.78	− 2.00
		O	30	CE	Lys	721	H-acceptor	3.25	− 1.00
		O	31	NZ	Lys	721	H-acceptor	2.98	− 5.50
**16**	− 7.61	O	19	OG1	Thr	766	H-acceptor	2.98	− 0.70
		O	20	CD	Lys	721	H-acceptor	3.67	− 0.20
**17**	− 10.90	O	33	OD2	Asp	831	H-donor	3.33	− 0.80
		O	34	OD2	Asp	831	H-donor	2.72	− 3.30
		O	28	OG1	Thr	766	H-acceptor	2.92	− 0.50
		O	32	N	Cys	773	H-acceptor	3.56	− 0.20

As revealed from Table 2, the sesterterpenoid derivatives phorbaketal A acetate **12** and secoepoxy ansellone A **13** together with mycalol-522**17** showed the best binding energy with EGFR that surpassed the co-crystallized ligand **AQ4**. They showed − 9.90, − 9.94 and − 10.90 Kcal/mol, respectively in comparison to -9.52 kcal/mol of **AQ4**. Moreover, all the derivatives except **4, 6, 11-14** and **17** managed to form one or more hydrogen bonds with the crucial Lys721 with an average bond length of 3.0 Å. Consequently, the achieved binding to Lys721 might hinder its function in activating EGFR as explained earlier. Furthermore, both phorbaketal A **11** and its acetate derivative **12** formed H-bonds with the hinge residue Met769 in a similar way to **AQ4** with average distance of 3.15 Å. On the other hand the diterpene derivative hamigeran L** 1** founded a H- bond of 2.82 Å length with the crucial Gln767 in resemblance to **AQ4**. In the same context both acetate moieties of **12** interacted with Asp831 and Met769 in addition to two H-bonds formation between the oxygen of the basic sesterterpenoid skeleton with Gly772 and Cys773 (Fig. 7a, b). Comparatively, the alkene terminus of secoepoxy Ansellone A**13** demonstrated hydrophobic interaction with Phe699 and hydrogen bond formation with Thr766 by its cyclohexenone carbonyl moiety of 3.08 Å length (Fig. 7c, d). In contrast, the hydroxyl groups of the open chain mycalol-522**17** fashioned four hydrogen bonds with Thr766, Cys773 and Asp831 with an average distance 3.0 Å (Fig. 8a). It was observed that the high molecular weight and the long carbon chain length of **17** managed to fill the active site of EGFR which consolidated its orientation with the formed bonds as illustrated in Fig. 8b. On the other hand, the acetate terminus and neighboring hydroxyl group of the sterol derivative phorbasterone D **10** revealed of five hydrogen bonds with the crucial Lys721, Thr830, Asp831 and Glu738 for optimum positioning inside the active site of EGFR (Fig. 9a). Similarly the carbonyl moiety of the octahydronaphthalene ring and its nearby hydroxyl group of the sesterterpenoid derivative ansellone G **15** formed multiple hydrogen bonds with Lys721 and Asp831 (Fig. 9b).

## Conclusion

The crude extract of *Hemimycale* sp. sponge was tested for its cytotoxic activity against various cell lines; only the breast cancer cell line showed promising activity, particularly after forming total extract loaded niosomes, with an IC_50_ value of 44.35 ± 0.011128 µg/ml with no cytotoxic effects on normal cell line, implying selectivity for breast cancer cell line and potentiality for enhancing the activity by utilizing niosomes as a nanotechnology-based drug delivery approach. The chemical profiling of crude extract resulted in the dereplication of seventeen compounds of various classes such as diterpenes, sesterterpenes, sterols, and others. The cytotoxic activity against breast cancer cell lines was then explained using molecular docking simulation, which revealed that the sesterterpenoid derivatives phorbaketal A acetate (**12**) and secoepoxyansellone A (**13**) in combination with mycalol-522 (**17**) had the best binding energy with the Epidermal Growth Factor Receptor tyrosine kinase (EGFR). *Hemimycale* sp. Sponge is a rich source of biologically active components that has the potential to be a promising drug candidate.

## Data Availability

All data are available in the manuscript.
